# Axillary Artery Injury Encountered During Chest Wall Tumor Resection

**DOI:** 10.7759/cureus.68144

**Published:** 2024-08-29

**Authors:** Yukio Umeda, Kiyohiko Hagiwara, Shinsuke Matsumoto

**Affiliations:** 1 Cardiovascular and Thoracic Surgery, Gifu Prefectural General Medical Center, Gifu, JPN

**Keywords:** iatrogenic injury, thoracic surgery, chest wall tumor, axillary artery, vascular injury

## Abstract

Axillary artery injuries are rare because of their anatomy but are sometimes fatal because of the difficulty of obtaining vascular integrity. We report a 50-year-old patient with an iatrogenic axillary arterial injury that occurred during the resection of a chest wall tumor. The injury occurred during an incision of the intercostal muscle along the superior margin of the second rib. Following primary hemostasis achieved by forceps and amputation of the pectoralis minor muscle, the injury site was exposed sufficiently and successfully repaired by a vascular surgeon. This successful case provided valuable insight into strategies, primary hemostasis, and subsequent revascularization for an intraoperative vascular injury.

## Introduction

The axillary artery is anatomically defined as the artery that runs between the lateral border of the first rib and the inferior border of the teres major muscle. Several cases of axillary artery injuries have been reported in association with orthopedic conditions such as shoulder dislocation or humeral fracture, as well as in particular trauma such as gunshot injury, bomb blast, and stabbing [1-4]. Axillary artery injuries are sometimes lethal due to the difficulty in approaching the artery and the lack of standardized reparative strategies [5].

We report a case of an open penetrating injury of the axillary artery during chest wall tumor resection. To the best of our knowledge, this is the first successfully treated case of iatrogenic axillary artery injury besides those related to orthopedic procedures.

The present case provides valuable insights for sufficient exposure of the injured site and subsequent repair. We believe that sharing this case will contribute to the establishment of standardized strategies for these rare vascular injuries.

## Case presentation

**Figure 1:**
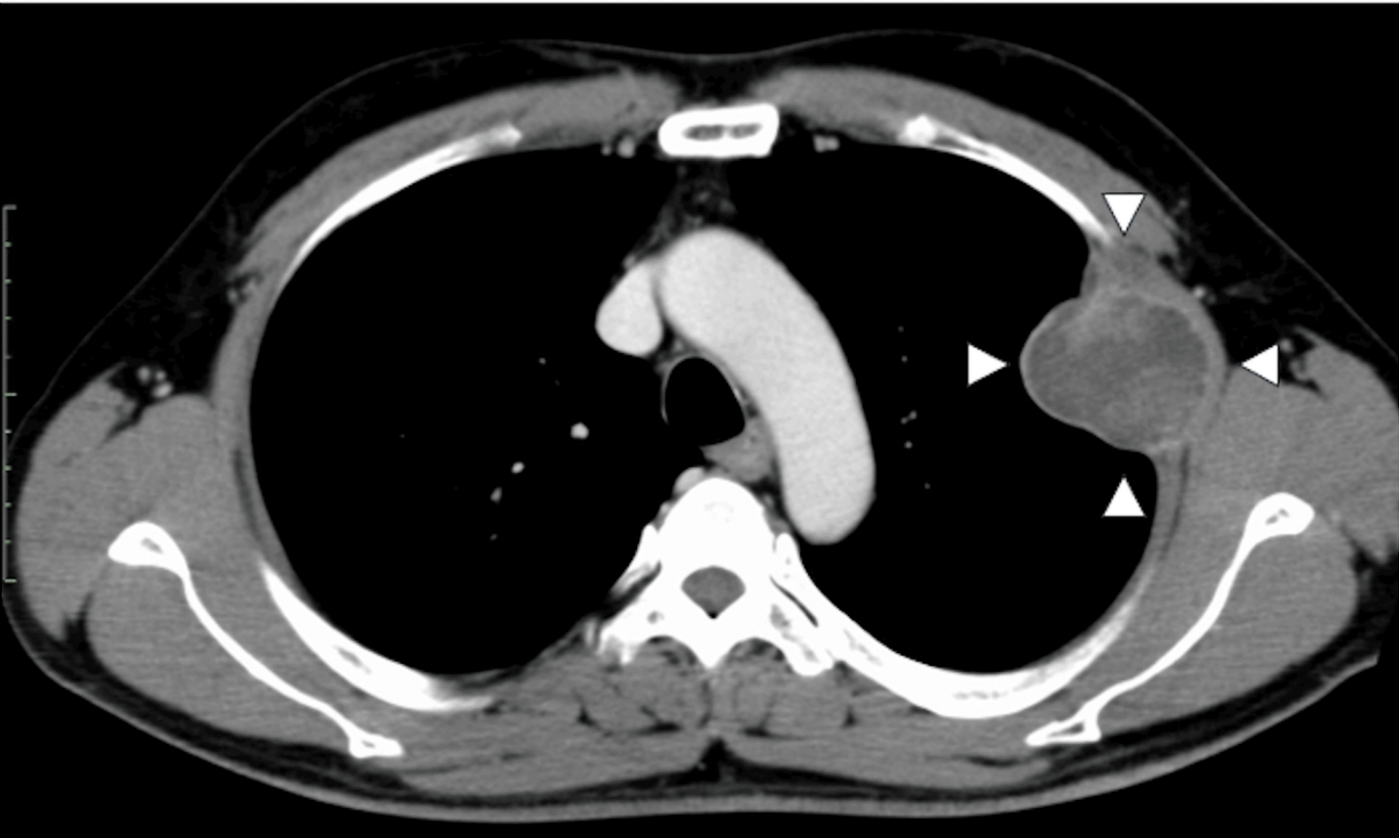
Preoperative CT scan Arrowheads indicate the tumor located inside and outside the thoracic cavity. CT: computed tomography

A 50-year-old male was referred to our hospital for a chest wall tumor detected by a CT scan. The patient underwent a CT scan for an abnormal shadow on the chest X-ray performed as a routine physical examination. He had no particular medical history. The tumor (63 x 60 x 52 mm) was located inside and outside of the left chest wall between the second and third intercostal spaces (ICS) and partially involved the left upper lobe of the lung (Figure 1).

A biopsy under ultrasonography revealed the tumor was undifferentiated sarcoma. An en bloc tumor resection was planned, which included resection of the second to fourth ribs, serratus anterior muscle, pectoralis minor muscle, and an upper lobectomy.

The surgical procedure was performed in a right lateral position by a thoracic surgeon and a thoracic and cardiovascular surgeon under epidural and general anesthesia with a dual-lumen endotracheal tube. The second, third, and fourth ribs ventral to the tumor were transected through a fourth ICS thoracotomy. The intercostal muscle of the first ICS was incised with an electrocautery scalpel along the upper margin of the second rib from the outside of the chest wall while observing from the inside via a thoracoscope. Massive bleeding through the first ICS was observed from inside the thoracic cavity at the exact moment when the electrocautery was directed laterally, which interfered with the medial border of the pectoralis minor muscle (Video 1).

**Video 1:** Axillary artery injury during the incision of the intercostal muscle

**Figure 2:**
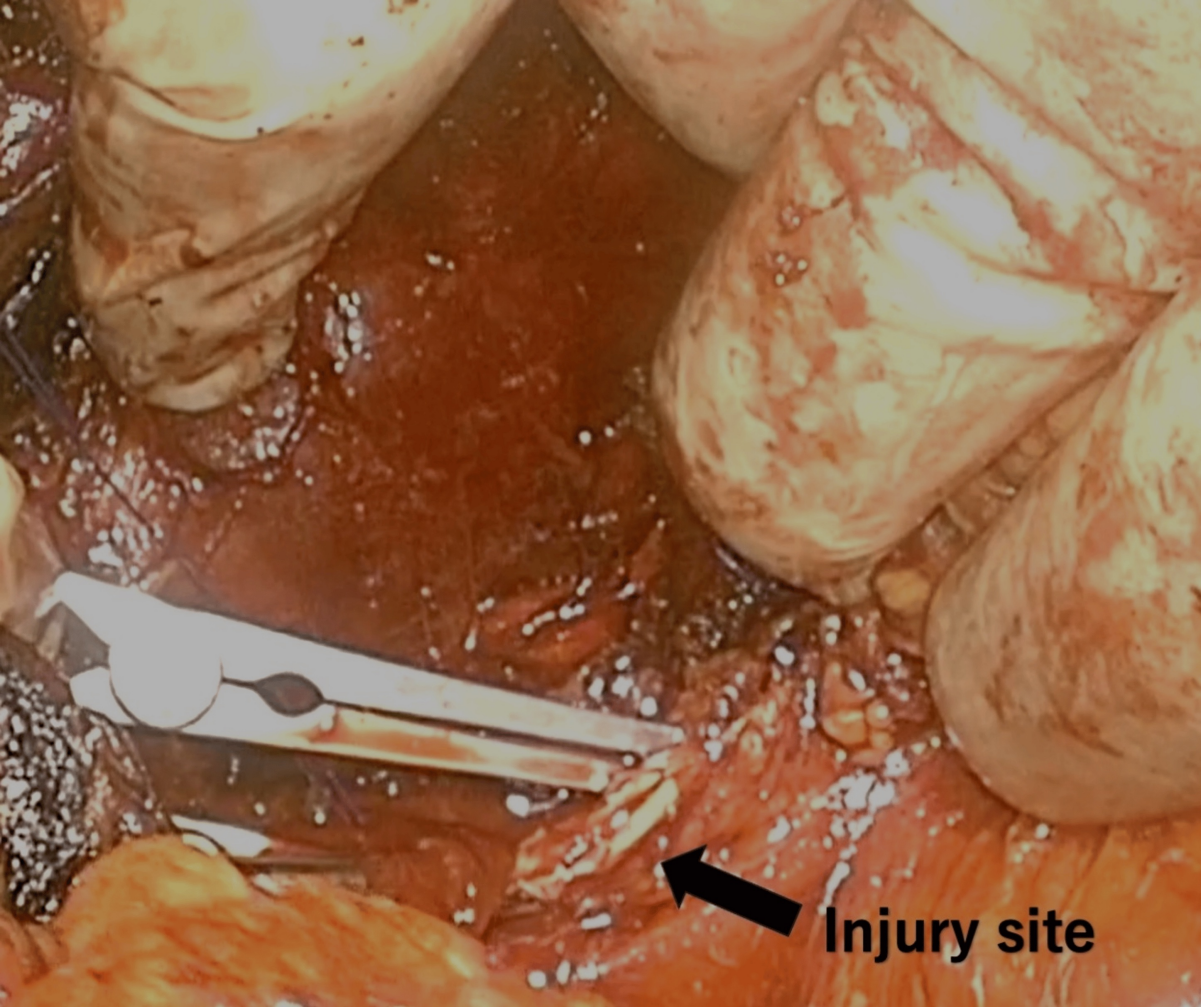
Injured site of the axillary artery Caudal wall of the left axillary artery was longitudinally incised.

The initial skin incision was extended while applying manual compression around the medial border of the pectoralis minor muscle. Following the exposure of the area between the pectoralis major muscle and the chest wall using a retractor, the manual compression site could be identified. The axillary vein was completely transected, which was closed with 4-0 Prolene (Ethicon Inc., Johnson & Johnson Company, New Brunswick, NJ, USA). The caudal wall of the axillary artery sustained a longitudinal injury (Figure 2, Video 2).

**Video 2:** Exposure of the injured site and temporary clamping of the axillary artery at the injured site

After temporary clamping at the injury site using forceps, the pectoralis minor muscle was transected near the scapular attachment site for exposure and in clamping the distal and proximal portions of the axillary artery, away from the injury, respectively. The injury site of the axillary artery was trimmed and repaired with interrupted 5-0 Prolene sutures (Ethicon Inc., Johnson & Johnson Company, New Brunswick, NJ, USA) (Video 3).

**Video 3:** After transection of the pectoralis minor muscle and exposure of the distal and proximal of the injury site, the axillary artery was trimmed and repaired

This procedure for obtaining hemostasis and repair was performed without heparinization. The procedure was carried on after confirming that there was no difference in blood pressure readings in the bilateral upper extremities. Transection of the second, third, and fourth ribs, dorsal to the tumor, and left upper lobectomy were performed. Thereafter, the tumor was removed en bloc. The chest wall was reconstructed with an ePTFE membrane (0.1-mm-thick, PRECLUDE®︎‎ Pericardial Membrane, W.L. Gore & Associates, Inc., Flagstaff, AZ, USA) (Video 4).

**Video 4:** Subsequent left upper lobectomy and reconstruction of the chest wall

**Figure 3:**
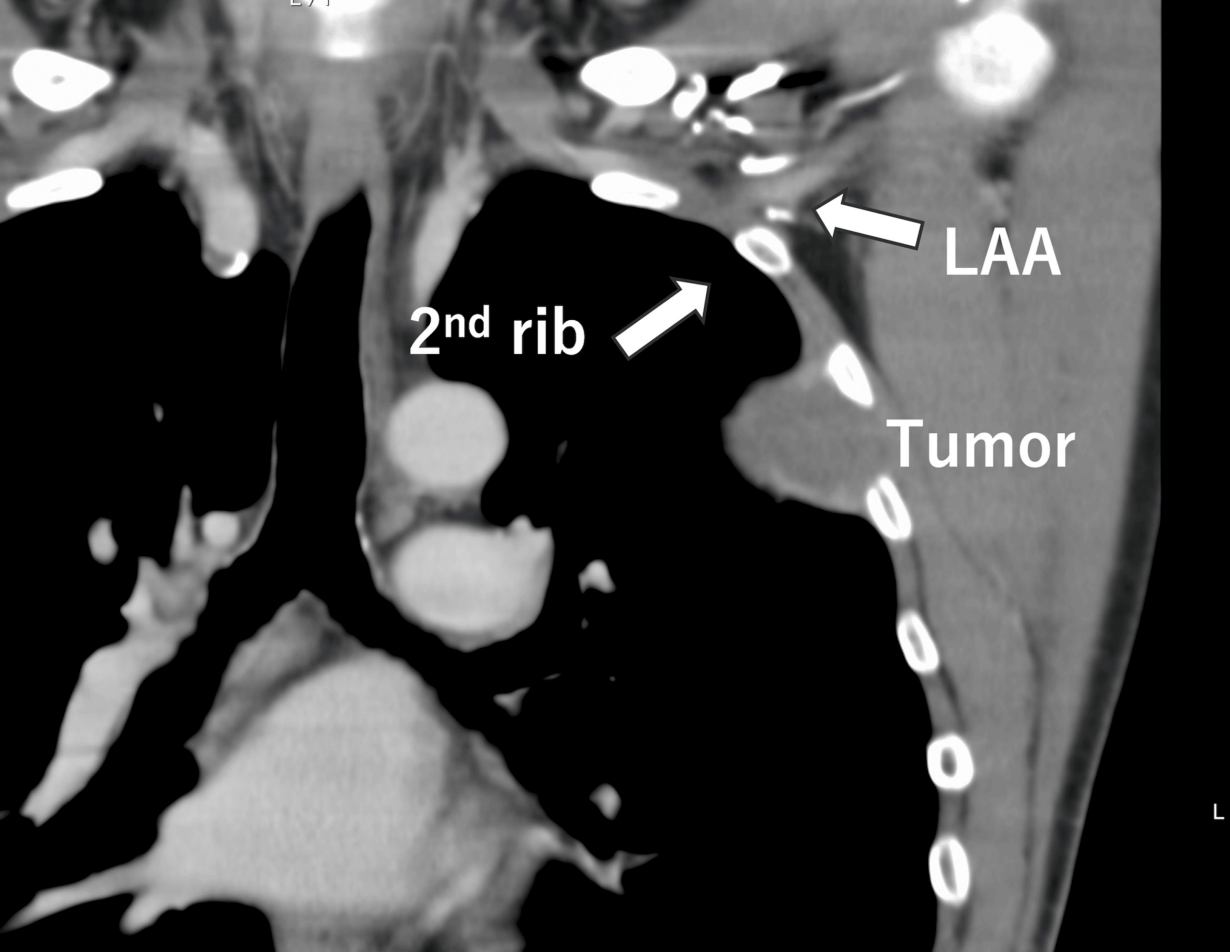
Retrospective review of the preoperative CT The LAA runs close to the superior border of the second rib. LAA: left axillary artery, CT: computed tomography

The operative time was three hours and 53 minutes, and the total blood loss was 1,980 ml, requiring the transfusion of six units of packed red blood cells. Retrospectively, preoperative CT showed that the axillary artery/vein ran very close to the superior border of the second rib (Figure 3).

The patient was discharged from the hospital on the 10th postoperative day, with no difference in blood pressure readings in the upper extremities, no neurological abnormality in the left upper extremity, and no significant stenosis confirmed by a postoperative enhanced CT scan. After discharge, radiation therapy (50 Gy) was added, and the patient is alive 6 years after surgery without recurrence.

## Discussion

Injury to the axillary artery is rare because it is protected by the clavicle and the pectoralis major and minor muscles. However, when injured, it may cause fatal bleeding. Its anatomy makes it difficult to approach immediately, and secondary injury to the adjacent brachial plexus during repair is also a concern [4,6]. Most of the axillary artery injuries reported to date in the civilian population are related to orthopedic conditions such as shoulder dislocation, humeral fracture, or scapulothoracic dissociation [1-4,7,8]. Furthermore, iatrogenic axillary artery injuries are rarely encountered, and their repair strategies should be shared [9-16]. We experienced a case of an axillary artery injury during chest wall tumor resection.

The cause of the axillary artery injury in the present case may have been due to the handling of the electrocautery during the incision of the first ICS. An excision of the pectoralis minor muscle from the coracoid process should have been performed to avoid a potential injury prior to the intercostal incision.

Currently, endovascular repair as well as open repair is an option for axillary artery injuries, and several reports have focused on the usefulness of endovascular repair [17,18]. On the other hand, Shih et al. reported a case of stent separation related to moving the shoulder joint after endovascular repair. In that case, the patient sustained a pulseless electrical activity arrest due to hemorrhagic shock. Complications related to endovascular repair, particularly to highly movable vessels such as the axillary artery, should also be carefully considered [19].

With respect to iatrogenic injuries, i.e., cases with vascular occlusion related to injury, endovascular repair has been performed recently [9,12], while open repair has been usually employed in cases with active bleeding related to the injury [10,11,14,16] or in cases of unsuccessful endovascular repair [9].

The approach for open axillary artery repair requires the mobilization of the pectoralis minor muscle due to its anatomy. To preserve the function of the pectoralis minor muscle, resection of the coracoid attachment would be generally performed and re-attached following the vascular repair [16]. In the present case, however, the pectoralis minor muscle was amputated at its attachment to the coracoid, as initially planned, and this also shortened the time to repair. Depending on the extent and the mechanism of injury, primary repair, end-to-end anastomosis, interposition, bypass, etc. are considered. In this case, interposition or bypass with a saphenous vein graft was not feasible for the patient's position and cleanliness of the surgical field. We employed a primary repair to shorten the time and avoid heparinization.

Particularly in the case of iatrogenic vascular injury during surgery, a sequence of strategies with secure vascular clamping to control severe bleeding and adequate revascularization to restore function is crucial. Previous papers concerning iatrogenic axillary artery injury have reported either consulting a vascular surgeon while compressing the injured area to control bleeding or transferring the patient to a facility where vascular repair could be performed after clamping the injury site. On the other hand, Takase et al. reported a case of subclavian artery injury during a left upper lobectomy [20]. A cardiovascular surgeon was consulted to repair the injury, but at the time of the consultation, the cardiovascular surgeon was informed that the site of the injury was unknown. A vascular repair was performed under hypothermic extracorporeal circulation. However, the patient died as a result of prolonged bleeding, probably accelerated by hypothermia. As in this report, recognition of the site, mode of injury, and sharing of precise information with the surgeon prior to the vascular repair are considered critical. Otherwise, even if extracorporeal circulation with or without hypothermia is not necessary, cardiovascular surgeons who are consulted may use it as a safety strategy beyond what is necessary if the site or mode of injury is unknown.

In the present case, the procedure was performed by a thoracic surgeon and a thoracic and cardiovascular surgeon, and the injured site and the mode of injury were recognized by the surgeon prior to the repair, which allowed for rapid repair. In fact, because the injured site was recognized in the first segment of the axillary artery, after primary clamping near the injury site, resection of the pectoralis minor muscle was performed to ensure a sufficient surgical field for repair. Sufficient dissection was performed to clamp the distal and proximal portions of the axillary artery away from the injury site, allowing definitive repair of the artery. These procedures were executed without heparinization, as rapid vascular clamp and repair were deemed feasible. This was advantageous in reducing the amount of blood loss during the subsequent rib resection, upper lobectomy, and reconstruction of the chest wall.

## Conclusions

We experienced a case of axillary artery injury during chest wall tumor resection. Axillary artery injury is a rare condition, and no standardized treatment strategy exists. Even in cases of massive hemorrhage, we often strive to achieve hemostasis and secure blood flow at the same time. However, performing hemostasis and revascularization in a step-by-step fashion, as in this case, would be a safe and reproducible strategy that strives for a favorable outcome.
